# Alternatives to Ciprofloxacin Use for Enteric Fever, United Kingdom

**DOI:** 10.3201/eid1405.071184

**Published:** 2008-05

**Authors:** E. John Threlfall, Elizabeth de Pinna, Martin Day, Joanne Lawrence, Jane Jones

**Affiliations:** * Health Protection Agency, London, UK

**Keywords:** Enteric fever, drug resistance, ciprofloxacin, cephalosporins, azithromycin, letter

## Abstract

Alternatives to Ciprofloxacin Use for Enteric Fever, United Kingdom

**To the Editor:** In cases of typhoid and paratyphoid fever, it is often necessary to commence treatment before the results of laboratory sensitivity tests are available. It is therefore important to be aware of optional drug therapies available because some organisms may be resistant to key antimicrobial drugs. For typhoid and paratyphoid, ciprofloxacin has become the first-line drug of choice since the widespread emergence and spread of strains resistant to chloramphenicol, ampicillin, and trimethoprim (*1*).

The Laboratory of Enteric Pathogens (LEP) of the Health Protection Agency of England and Wales is the reference center for *Salmonella enterica* serovars Typhi and Paratyphi A for the United Kingdom; as such, this laboratory receives isolates from all cases of infection. Isolates are screened by breakpoint for resistance to antimicrobial drugs at the following levels: chloramphenicol, 8 mg/L; ampicillin, 8 mg/L; trimethoprim, 2 mg/L; ciprofloxacin, 0.125 mg/L (decreased susceptibility); and 1.0 mg/L (high-level resistance), ceftriaxone, 1 mg/L, and cefotaxime, 1 mg/L. The levels for testing for resistance to chloramphenicol, ampicillin, trimethoprim, ceftriaxone, and cefotaxime correspond to internationally accepted therapeutic levels for these antimicrobial agents. In contrast, the levels for ciprofloxacin (0.125 and 1.0 mg/L) have been chosen after observations of treatment failures at levels when used at below the expected recommended serum concentrations (*2*,*3*). Since 2005, a proportion of isolates exhibiting decreased susceptibility and high-level resistance to ciprofloxacin have been tested for resistance to azithromycin by Etest (AB Biodisk, Solna, Sweden), using drug-sensitive strains of *S.* Typhi and *S.* Paratyphi A as controls.

From January 2001 through December 2006, LEP reported 1,215 cases of *S.* Typhi infection and 1,274 cases of *S.* Paratyphi A infection. Of these, ≈60% (1,493) reported recent travel abroad; India and Pakistan were the most frequently visited countries (*4*). Other cases were associated with persons who had a history of such travel, but the numbers involved were difficult to document accurately because of underreporting of foreign travel and other communication problems.

For *S.* Typhi, the occurrence of isolates resistant to ciprofloxacin at 0.125 mg/L increased from 60 (35%) of 170 in 2001 to 169 (70%) of 240 cases in 2006, with 4.8 (2%) of isolates in 2006 resistant at 1.0 mg/L (Table). The corresponding figures for *S*. Paratyphi A were 58 (25%) of 232 cases in 2001, rising to 84% in 2004, with an incidence of 73% in 2006; 9% of these were resistant to ciprofloxacin at 1.0 mg/L (Table). Moreover, in 2006, 56 isolates of *S.* Typhi (23% of total) exhibited resistance to chloramphenicol, ampicillin, and trimethoprim, 54 (96%) were also resistant to ciprofloxacin at 0.125 mg/L. When tested for resistance to ceftriaxone and cefotaxime, none of the isolates (either *S.* Typhi or *S.* Paratyphi A) were resistant at 1.0 mg/L.

**Table:** Incidence of resistance/decreased susceptibility to key antimicrobial agents in isolates of *Salmonella enterica* serovars Typhi and Paratyphi A, United Kingdom, 2001–2006*

Year	No. studied	% *S*. Typhi resistant to					No. studied	% *S*. Paratyphi A resistant to				
		C	A	Tm	Cp_L_	Cp_H_		C	A	Tm	Cp_L_	Cp_H_
2001	170	24	23	23	35	0	232	28	27	27	23	2
2002	150	18	17	17	35	1	149	10	9	10	39	3
2003	218	20	20	21	43	1	177	17	18	17	65	12
2004	215	23	23	24	47	2	221	5	5	5	70	14
2005	222	29	29	29	62	2	217	7	7	7	60	12
2006	240	23	24	24	68	2	278	2	3	2	64	9

*C, chloramphenicol; A, ampicillin, Tm, trimethoprim, Cp_L_, ciprofloxacin MIC 0.25–1.0 mg/L; Cp_H_, ciprofloxacin MIC >1.0 mg/L. No isolates exhibited resistance to ceftriaxone or cefotaxime; of 50 *S*. Typhi and 40 *S.* Paratyphi A isolated in 2005 and 2006, the MIC to azithromycin by E test (AB Biodisk, Solna, Sweden) was not greater than 8 mg/L for *S.* Typhi and 12 mg/L for *S.* Paratyphi A, which corresponds to those of drug-sensitive controls of the respective serotypes.

Although the levels of resistance to ciprofloxacin were for the most part below that regarded as therapeutic (MIC 0.25–1.0 mg/L), at least 21 treatment failures have been documented since 2005. These findings demonstrate that the efficacy of ciprofloxacin for first-line treatment of enteric fever in the United Kingdom has been seriously jeopardized. In cases of treatment failures, commonly used alternative antimicrobial agents have included third-generation cephalosporins such as ceftriaxone. The macrolide antimicrobial azithromycin is also being increasingly used, particularly for patients with hypersensitivity to penicillins (*5*). With this in mind, 50 *S.* Typhi and 40 S. Paratyphi A strains isolated from January 2005 through December 2006, which exhibited resistance to ciprofloxacin at 0.125 mg/L, were tested for resistance to azithromycin by Etest. Results indicated that none of the isolates of *S.* Typhi exhibited MICs >8 mg/L, which corresponded to the MIC to azithromycin of a drug-sensitive control strain of *S.* Typhi (range 4–8 mg/L, MIC_90_ 6 mg/L). For *S.* Paratyphi A, none of the isolates exhibited MICs >12 mg/L, corresponding to that of a drug-sensitive control strain of this serovar (range 6–12 mg/L, MIC_90_ 10 mg/L). Although there are no definitive data on resistance levels for azithromycin in relation to treatment of typhoid and paratyphoid, these findings suggest that resistance to this antimicrobial agent in terms of treatment efficacy has not yet been jeopardized.

These results indicate that the availability of effective antimicrobial agents for the treatment of typhoid and paratyphoid infection is becoming increasingly limited for patients in the United Kingdom. Nevertheless, despite the dramatic upsurge in the occurrence of strains with decreased susceptibility, ciprofloxacin still remains the drug of choice for many physicians. It is reassuring that in cases of treatment failure, third-generation cephalosporins such as ceftriaxone and macrolide antimicrobial agents such as azithromycin appear to be viable alternatives.
